# Barriers and Facilitators to Implementing Point‐of‐Care Ultrasound to Confirm Gastric Tube Placement in Preterm Infants: A Qualitative Study

**DOI:** 10.1155/jonm/3537619

**Published:** 2026-06-03

**Authors:** Yajing Wang, Jiong Zhou, Jiahua Zhang, Yunwei Li, Yalin Jing, Yuting Xie, Yao Chen, Ying Zhou, Jingjing Jiang, Shuju Feng

**Affiliations:** ^1^ Neonatal Intensive Care Unit, Peking Union Medical College Hospital, Chinese Academy of Medical Sciences & Peking Union Medical College, Beijing, China, cams.ac.cn; ^2^ Department of Pediatrics, Peking Union Medical College Hospital, Chinese Academy of Medical Sciences & Peking Union Medical College, Beijing, China, cams.ac.cn

**Keywords:** gastric tube, neonatal intensive care unit, point-of-care ultrasound, preterm, qualitative study

## Abstract

**Aim:**

To explore the barriers and facilitators influencing the use of point‐of‐care ultrasound (POCUS) for confirming the position of gastric tubes in preterm infants in the neonatal intensive care unit (NICU).

**Background:**

Many research studies indicated that POCUS is an effective and repeatable technique for monitoring the tip position of gastric tubes; however, the application rate of this technique among nurses in the NICU for confirming the position of gastric tubes in preterm infants remains low.

**Methods:**

Using a qualitative descriptive research design, we conducted interviews with direct care nurses (*n* = 8), frontline doctors (*n* = 2), and nursing leaders (*n* = 2) at a tertiary‐level hospital to explore the barriers and facilitators influencing nurses’ use of POCUS for confirming the position of gastric tubes in preterm infants.

**Results:**

The study identified two main themes: facilitators and barriers. The facilitators include the following six subthemes: adequate resource allocation and equipment; demonstrable safety and clinical efficacy; intrinsic motivation and professional development; supportive and innovative work environment; strong managerial and leadership support; and clear pathways for process optimization. The barriers also include six subthemes: deficiencies in competency development systems; complexity of neonatal clinical presentations; absence of institutional policies and standardized protocols; sociocultural and cognitive resistance; perceived legal and ethical risks; and unclear team collaboration dynamics.

**Conclusion:**

This study explored the main factors hindering and promoting the implementation of the POCUS for gastric tube position confirmation from the perspectives of direct care nurses, frontline doctors, and nursing leaders. Participants pointed out that there were issues with insufficient protection and support in areas such as training systems, institutional regulations, cognitive levels, and team collaboration. Therefore, it is necessary to formulate strategies and policies to address these obstacles.

## 1. Background

Preterm infants are newborns with a gestational age of less than 37 weeks. The estimated global preterm prevalence in 2020 was 9.9%, translating to 13.4 million preterm live births. China has 750,000 preterm infants, ranking fourth globally [1].

Since premature infants have not yet developed mature sucking and swallowing functions before reaching a corrected gestational age of 32–34 weeks, a nasogastric tube (NGT) or orogastric tube (OGT) is needed after birth for enteral feeding, drug administration, and gastric decompression [2, 3]. Currently, NGT/OGT insertion in preterm infants is often performed blindly, with intubation length calculated using either a surface measurement method or a modified weight‐to‐length formula [4, 5]. However, due to significant anatomical differences between preterm infants and adults, as well as rapid postnatal growth in weight and length, the above methods are prone to malposition. Studies have shown that 47.5%–59% of newborns have improper tube placement [6–8]. A gastric tube placed too shallowly may cause nausea, vomiting, aspiration pneumonia, and suffocation in preterm infants, while placement too deeply may damage the gastric mucosa, potentially leading to severe complications such as gastric perforation [8]. The placement of gastric tubes should vary depending on the clinical situation [3]. Accurate assessment of the placement of the gastric tube is one of the key factors in ensuring the safety of gastric tubes in premature infants.

At present, commonly used methods for determining gastric tube placement include bedside X‐ray, auscultation, bubble method, gastric fluid aspiration, and pH measurement [6]. Although bedside X‐ray is considered the gold standard verification method, its use should be limited because continuous verification is not feasible in clinical practice due to radiation exposure [9]. Other methods can only indicate that the gastric tube may be in the stomach, but cannot determine its precise location. Therefore, new methods are needed to confirm the placement of gastric tubes in premature infants and enable continuous verification.

Point‐of‐care ultrasound (POCUS), due to its rapid, repeatable, nonradioactive characteristics, has been widely applied in neonatal intensive care units (NICUs) for tracheal intubation placement and tip position assessment, central venous catheter placement, and gastric emptying time evaluation [10]. Additionally, a systematic review summarized the application of ultrasound in determining the placement of gastric tubes in newborns and children, noting that the estimated sensitivity of ultrasound ranged from 0.88 to 1.00 [11]. It suggested that ultrasound may be an important method for accurately identifying the placement of gastric tubes in preterm infants. However, the current application in preterm infants is still limited. Only two studies have quantitatively analyzed the accuracy of ultrasound in confirming the placement of NGTs in premature infants [12, 13]. Qualitative research methods focus on exploring the inner world of research subjects in depth, revealing underlying causes that are difficult to obtain through quantitative research. When information is scarce, qualitative research is more suitable for understanding phenomena of interest [14]. Therefore, this study aims to better understand the perceptions of direct care nurses, frontline physicians, and nursing leaders regarding the barriers and facilitators influencing nurses’ acceptance and use of ultrasound for gastric tube placement. The study results will contribute to the development of nursing leadership interventions, promote broader adoption of POCUS technology among nurses, enhance nursing quality, and ensure clinical safety for preterm infants.

## 2. Methods

### 2.1. Study Design and Setting

A descriptive qualitative research method was used to interview individuals face‐to‐face through a semistructured interview. The study followed the Consolidated Criteria for Reporting Qualitative Research (COREQ) Checklist, which is widely recognized in the literature and is recommended for the design and reporting of qualitative research. We recruited nurses from the NICU of a tertiary hospital in Beijing, northeastern China.

### 2.2. Participants

Based on the maximum variation sampling strategy, we selected interviewees according to their professional roles, years of experience, ultrasound operation expertise, and certification status. The sample size was determined by the principle of data saturation, where no new themes emerged. Nurses, nurse leaders, and doctors were eligible if they met the following criteria: (1) had work experience in the NICU for ≥ 3 years; (2) had participated in any ultrasound‐related skills training; and (3) had prior exposure to or basic training in ultrasound operation (e.g., lung ultrasound and central venous catheterization), including both certified practitioners and those who had completed operation training modules but were awaiting formal certification. Other inclusion criteria included good mental health (not having been clinically diagnosed with any psychiatric disorder that could potentially affect cognition or communication [e.g., major depression, anxiety disorders] during the study period), good memory (the ability to clearly and coherently recount key events and details of POCUS‐related clinical procedures performed within the past 6 months), and fluent language skills (the ability to engage in unimpeded daily and professional communication in Chinese [the study language] and clearly express one’s views and feelings). The exclusion criteria were participants who were unable to participate in interviews due to extended absences from their positions during the study period for various reasons (personal leave, sick leave, or maternity leave) or due to training‐related assignments.

### 2.3. Data Collection

From May to June 2025, the study collected data through in‐depth, face‐to‐face, semistructured interviews. A preliminary interview outline was established after clarifying the research questions and reviewing the existing literature. Based on this, three pilot interviews were conducted (not included in the formal interview content), followed by a further review of the outline by four experts, two of whom had experience in NICU nursing management and the other two had in‐depth research experience in the field of intensive care ultrasound. The interview outline consisted of eight questions, as detailed in Table 1.

**TABLE 1:** Outline of the interview.

Number	Questions
1	What is your opinion on the use of POCUS to confirm the position of a gastric tube in premature infants?
2	Please share your experiences and insights gained during the process of using POCUS to confirm the position of gastric tubes in premature infants. What challenges did you encounter during the procedure, and what insertion techniques did you employ?
3	What differences do you think exist between using POCUS to confirm the position of gastric tubes in premature infants and adults?
4	What is your understanding of the training requirements for NICU nurses regarding the use of POCUS to confirm the placement of gastric tubes in premature infants?
5	What kind of support do you think healthcare providers who use POCUS to confirm the placement of gastric tubes in premature infants would receive?
6	What do you think are the obstacles to the clinical application of this technology?
7	What do you consider to be the favorable factors influencing the clinical implementation of this technique?
8	Do you believe this technique is worth promoting? Why?
9	Do you have any other suggestions or opinions regarding the use of POCUS to confirm the placement of a gastric tube in premature infants?

The interviewers had received training in qualitative research interview techniques. All interviews were conducted face‐to‐face in quiet, private, independent meeting rooms to avoid any disturbances. Each interview began with greetings and an introduction to the purpose of the study, followed by a step‐by‐step progression according to the interview outline. The interviews were recorded in their entirety to avoid missing important information. At the same time, the interviewers recorded key points for data analysis. The interviews were conducted in Chinese, which is the native language of both the interviewers and the participants. No new themes emerged during the interviews, indicating that the data had reached saturation, and data collection was subsequently halted. All scheduled interviews were successfully completed without any cases requiring exclusion due to interruption.

### 2.4. Data Analysis

Within 24 h of the interview, the audio recordings were transcribed verbatim into text using Xunfei recording software. Two researchers listened to the recordings repeatedly, cross‐checked them, and fully transcribed the text data to ensure its accuracy and completeness. The data were then imported into NVivo 11.0 software for analysis and management. This study adopted Colaizzi’s seven‐step method. Two researchers discussed the inductively derived themes to reach consensus. If disparities arose, a third researcher analyzed the original materials and participated in the discussion to make the final decision. The text was then returned to participants to check its accuracy.

### 2.5. Ethical Considerations

This study was approved by the Ethics Review Committee of Peking Union Medical College Hospital, Chinese Academy of Medical Sciences (I‐23PJ942). Prior to obtaining written and verbal consent, researchers provided all participants with a detailed explanation of the study’s purpose and objectives and outlined the data collection methods. Participants agreed to participate in the study and consented to being recorded, while also signing a written informed consent form. The informed consent form emphasized that participation in the study was entirely voluntary. Participants were informed that they had the right to withdraw from the study at any time, whether before or during participation. Data protection safeguards were provided to participants at all stages of the study.

### 2.6. Rigor and Trustworthiness

This study adopted a collaborative analysis method, with two master’s degree researchers responsible for data organization and analysis, and data verification with interviewees to enhance the credibility of the research. During data analysis, the researchers directly quoted the interviewees’ original statements to enhance reliability. To enhance verifiability, the researchers retained all original materials (memorandums, interview transcripts, and process notes) for subsequent review. To enhance transferability, the study presented information details as comprehensively as possible.

## 3. Results

From May 2025 to June 2025, a total of 12 healthcare professionals were interviewed, including 8 direct care nurses (designated as N1–N8), 2 nursing leaders (designated as NL1 and NL2), and 2 physicians (designated as D1 and D2). We intentionally included healthcare professionals at different stages of training and application (including nurses who completed training but had not yet received formal certification). Participant characteristics are summarized in Table 2. A total of 12 subthemes were extracted, which were summarized into two core themes.

**TABLE 2:** Participant characteristics.

No.	Sex	Age	Education	Years in nursing	Staff titles	Years since obtaining ultrasound certification
N1	F	41	BS	19	Nurse in charge	2
N2	F	42	BS	19	Nurse in charge	7
N3	F	38	BS	15	Nurse in charge	7
N4	F	37	BS	14	Nurse in charge	3
N5	F	31	BS	8	Nurse in charge	2
N6	F	31	BS	8	Nurse in charge	3
N7	F	25	BS	3	Nurse practitioner	/^∗^
N8	F	23	BS	3	Nurse practitioner	/^∗^
D1	F	34	MD	8	Physician in charge	6
D2	F	34	MM	9	Physician in charge	7
NL1	F	49	BS	27	Associate Chief of Nursing	7
NL2	F	39	MSN	16	Associate Chief of Nursing	3

*Note:* N1–N8, Nurses 1–8; F, female.

Abbreviations: BS, Bachelor of Science; D1/D2, Doctor 1/Doctor 2; MD, Doctor of Medicine; MM, Master of Medicine; MSN, Master of Science in Nursing; NL1/NL2, Nurse Leader 1/Nurse Leader 2.

^∗^“Uncertified” status refers to having completed foundational training but not yet passed final competency assessments or authorization. All participants met the inclusion criterion of “(2) participated in any ultrasound‐related skills training.”

### 3.1. Theme 1: Facilitators

#### 3.1.1. Adequate Resource Allocation and Equipment

Participants strongly emphasized that accessible, appropriate, and high‐quality ultrasound equipment is fundamental for successful integration into the NICU workflow. Key aspects include device availability, portability, image quality, and probe suitability for neonates.

“The accessibility and portability of the ultrasound machine are crucial. If each patient room had its own dedicated machine, eliminating the need to fetch one from a central equipment room, it would significantly enhance feasibility… I’ve seen handheld portable ultrasound devices; having those would be even more convenient, though departmental budget constraints might be a barrier to acquiring them.” (N4)

“Modern ultrasound equipment with better resolution is essential, particularly for reliably identifying the mist sign. Older machines in our unit often produced very fuzzy, indistinct images, making interpretation difficult and unreliable.” (L2)

“Technological advancements are promising, with newer ultrasound systems offering micro‐convex or high‐frequency linear probes specifically designed for neonatal anatomy. These appropriately sized probes allow for better contact and maneuverability on tiny infants, facilitating more accurate imaging.” (N2)

#### 3.1.2. Demonstrable Safety and Clinical Efficacy

Nurses highlighted a significant advantage: Implementing ultrasound confirmation does not impose additional direct costs on families compared to traditional confirmation methods, while simultaneously offering a superior safety profile. This creates a compelling value proposition for parental acceptance and institutional support.

“This technique doesn’t incur extra charges for parents beyond the standard costs associated with traditional gastric tube placement and confirmation. There are no additional disposable supplies required. Crucially, it enhances infant safety, making it a practice that parents can readily understand and support.” (N5)

Multiple participants reported observable benefits during practice, including reduced procedural time, decreased infant distress and facial discomfort, and lower incidence of complications like vomiting, retching, and abdominal distension. This directly enhances the infant’s experience and clinical stability.

“In practical application, we consistently observe reduced insertion times. Infants exhibit less agitation, display fewer signs of facial discomfort, and experience lower rates of nausea, vomiting, and abdominal distension.” (N1)

The real‐time visualization capability allows for dynamic adjustment of tube tip position based on the specific clinical indication (e.g., gastric decompression, lavage, and feeding), moving beyond a one‐size‐fits‐all approach toward individualized, optimized care.

“Different clinical scenarios–whether for gastric decompression, lavage, or enteral feeding–necessitate precise positioning of the gastric tube tip… Ultrasound guidance enables real‐time visualization and adjustment based on the specific therapeutic need, significantly elevating the quality and precision of nursing care.” (N2)

“I firmly believe this is a valuable technique. It can effectively reduce complications such as intra‐esophageal looping or coiling of the tube, and aspiration events, providing a higher level of protection for these vulnerable infants.” (D2)

#### 3.1.3. Intrinsic Motivation and Professional Development

Nurses expressed strong internal drivers, including personal interest, a sense of professional responsibility, and the desire for career advancement, fueling their willingness to invest significant time and effort into mastering this skill. Acquiring this competency fosters a sense of accomplishment, enhances job satisfaction, and provides a clear pathway for professional growth and recognition.

“Mastering this technique adds another valuable skill to my repertoire, generating a strong sense of personal achievement. It makes my work feel more meaningful and engaging.” (N5)

“Beyond acquiring a practical skill… this represents a viable and novel area for nursing research. Engaging in this field holds tangible benefits for professional development and career progression, including opportunities for publication and meeting criteria for promotion.” (N8)

#### 3.1.4. Supportive and Innovative Work Environment

A positive unit culture characterized by peer mentorship, visible leadership support, and a collective drive for continuous learning acts as a powerful catalyst. When nurses observe colleagues, particularly senior nurses and leaders, embracing and excelling in the technique, and receive active encouragement and guidance, their own motivation and confidence increase.

“The learning environment and culture within our unit are excellent. Several senior nurses demonstrate strong professional identity and mastery; we junior nurses actively look up to them as role models and mentors.” (N7)

“There’s a palpable ethos of professional growth among colleagues in the department. There’s a collective enthusiasm for actively learning and adopting new evidence‐based technologies and practices.” (N6)

#### 3.1.5. Strong Managerial and Leadership Support

Nurses in leadership positions (e.g., Nurse Managers, Clinical Nurse Specialists) expressed unequivocal endorsement of the technology. They emphasized the strategic importance of supporting implementation through concrete actions, such as optimizing staffing models to include certified nurses, allocating resources for training, and fostering interprofessional collaboration.

“Implementing this technique is not just beneficial, it’s necessary, and I am fully committed to supporting it… Fundamentally, it’s an efficiency measure–it can conserve nursing time and reduce workload burdens in the long run… A practical strategy involves establishing an ‘Ultrasound Core Group,’ comprising nurses who have undergone specialized training and obtained certification. This ensures the presence of an expert resource nurse within each team and on every shift, providing peer support and guidance.” (L1)

“We aim to actively promote and facilitate multidisciplinary collaboration. This includes organizing educational sessions featuring experts from Neonatology, Pediatric Critical Care, and Diagnostic Ultrasound to deepen understanding and refine protocols across disciplines.” (L2)

#### 3.1.6. Clear Pathways for Process Optimization

Participants offered concrete, actionable recommendations for refining the procedure and its integration into practice. These suggestions focused on enhancing safety, standardizing training, improving simulation opportunities, and addressing the unique physiological needs of neonates during the procedure.

“Thermoregulation is paramount for preterm infants… Ultrasound‐guided placement requires close collaboration between nurse and physician to ensure continuous physiological monitoring and minimize heat loss… Specific thermoprotective measures must be rigorously implemented, including pre‐warming ultrasound gel and strictly limiting the duration the incubator portholes are open during probe manipulation.” (D1)

“Access to high‐fidelity simulation models for deliberate practice would be invaluable. Practicing extensively on simulators before performing the procedure on actual neonates would significantly improve operator dexterity, reduce procedural time, and increase accuracy, thereby enhancing patient safety during the learning curve.” (N2)

“Achieving consistent competency across practitioners requires a strong emphasis on standardized, uniform training curricula coupled with rigorous, objective assessment methods. Ensuring all certified nurses reach and maintain a demonstrable, high level of proficiency is crucial for safe and effective implementation.” (D2)

### 3.2. Theme 2: Barriers

#### 3.2.1. Deficiencies in Competency Development Systems

Current training programs were universally reported as inadequate and lacking specificity for the neonatal population. The nascent stage of ultrasound application for neonatal gastric tube placement confirmation in clinical practice directly contributes to this gap. Training content fails to comprehensively address the unique challenges encountered in the NICU, hindering both the initial adoption and subsequent refinement of the technique by nurses.

“There is currently a lack of standardized training for this technique. While some individuals possess certification, it doesn’t resolve all operational issues encountered in practice. For instance, utilizing ultrasound to confirm tube position in extremely low birth weight infants isn’t being implemented yet due to concerns about potential harm.” (N3)

“The existing ultrasound‐guided gastric tube placement protocols are designed for adults, not neonates. We are still in the pioneering phase, lacking substantial experience, and require further exploration and adaptation for this vulnerable population.” (D1)

Nurses highlighted that the predominant training model, often led by ultrasound manufacturers focusing on machine operation or delivered by physicians for physician practice, offered low relevance and guidance for nursing‐specific applications. This diminished nurses’ sense of engagement and ownership of the skill.

“The training primarily involves ultrasound manufacturers explaining machine usage… In clinical settings, ultrasound operation is predominantly performed by physicians. There is a distinct absence of training specifically tailored for nurses’ needs and scope.” (N4)

The absence of a tiered or competency‐based teaching approach was a significant hurdle. Training programs were often monolithic, failing to cater to the diverse learning paces, prior experience levels, and individual learning capacities of nurses. This “one‐size‐fits‐all” approach prevented nurses from achieving proficiency commensurate with their background and hindered targeted skill development.

“Individual learning capabilities vary significantly. Even with strong motivation and diligent effort, nurses with inherent learning limitations may struggle to achieve competence. Without differentiated support, they may plateau or become discouraged despite their desire to master the skill.” (L2)

Obtaining formal ultrasound operation certification often entailed substantial out‐of‐pocket expenses for nurses. This financial barrier created hesitation and reluctance, effectively preventing qualified and willing nurses from gaining the necessary credentials to practice independently.

“Participating in training offered by the Critical Care Ultrasound Association requires self‐funding–around 2000 CNY. This is a considerable sum, and the department does not reimburse it. Currently, departmental policy mandates possessing this specific certificate to perform the ultrasound confirmation procedure and interpret the images. Consequently, even if we desire to utilize ultrasound for gastric tube placement, we are prohibited from doing so without this costly credential.” (N7)

#### 3.2.2. Complexity of Neonatal Clinical Presentations

Nurses consistently emphasized that intrinsic patient factors significantly amplify the technical difficulty compared to adult practice. Variables such as very low birth weight, extreme prematurity, minute gastric fluid volumes (e.g., small milk feeds), and the presence of comorbidities (like abdominal distension or organomegaly) profoundly impact both the procedural execution and the interpretation of ultrasound images.

“After placing the gastric tube via conventional methods and confirming its position within the stomach, we need to inject at least 10 milliliters of fluid to reliably observe the ultrasonic mist sign. Therefore, only premature infants who have reached this fluid intake threshold can undergo this procedure more safely.” (N1)

“Successful tube placement is heavily affected by the infant’s weight. Placement in very low birth weight infants presents inherent difficulties… Conditions like significant abdominal gas or hepatosplenomegaly can substantially obscure the view, making it challenging to locate the tube tip accurately.” (N3)

“Compared to placing gastric tubes in adults, the neonatal procedure is inherently more complex. Neonates are prone to agitation, have much narrower esophageal lumens, and demand a higher level of skill in image acquisition and interpretation.”(D2)

#### 3.2.3. Absence of Institutional Policies and Standardized Protocols

A critical barrier is the absence of official institutional or regulatory guidelines delineating the scope of practice for nurses regarding ultrasound use for gastric tube confirmation. This ambiguity directly impedes the formal recognition of nurse competency and the credentialing for this specific task.

“Currently, there are no established policies specifying what type of certification authorizes a nurse to utilize ultrasound for determining gastric tube tip position… In fact, there isn’t even a formal mandate requiring a certificate specifically for ultrasound image interpretation in this context, as we are not required to generate formal diagnostic reports. However, departmental policies often fill this void inconsistently.” (N6)

Nurses expressed a lack of confidence stemming from the nonexistence of formally established, evidence‐based nursing procedures or clinical guidelines specifically for ultrasound‐guided gastric tube placement and confirmation in neonates. The absence of clear, step‐by‐step protocols leaves nurses uncertain about best practices.

“We lack standardized work protocols and established nursing routines for this procedure. Without formalized operational guidelines and nursing standards, it’s unclear how to perform the procedure correctly and safely. This ambiguity undermines our confidence.” (N3)

#### 3.2.4. Sociocultural and Cognitive Resistance

Resistance stems from varying levels of awareness and acceptance among key stakeholders—including some nurses, physicians, and parents—regarding the demonstrable advantages of ultrasound over traditional methods (like auscultation and pH testing). Skepticism or lack of understanding from these groups hinders wider adoption.

“Since ultrasound confirmation of gastric tube placement is still largely exploratory in our setting… some parents perceive it as experimental and express concerns that ‘you might be using our child as a test subject’ leading to refusal of consent.” (N3)

“We sometimes encounter a lack of physician support. Some doctors express concerns that manipulating the ultrasound probe over the infant’s abdomen to locate the image might potentially disturb or harm the fragile infant.” (N1)

“A degree of cognitive conservatism exists among some nurses. They may be resistant to continuously adopting new technologies, perceiving this method as unnecessarily cumbersome or time‐consuming compared to familiar routines.” (L2)

#### 3.2.5. Perceived Legal and Ethical Risks

The unclear demarcation of responsibility and accountability for ultrasound‐guided procedures between nurses and physicians creates apprehension. Nurses fear potential medicolegal consequences if an error in image interpretation leads to a complication (e.g., perforation), especially in the absence of explicit protocols defining roles and liabilities.

“Even after obtaining my ultrasound certification, I hesitate to operate independently. Who bears the responsibility if an image misinterpretation results in a perforation? The Nursing Department advises ‘follow the physician’s order’, but physicians might say ‘you performed it, you are responsible’. This ambiguity surrounding accountability creates significant anxiety and discourages practice.” (N7)

#### 3.2.6. Unclear Team Collaboration Dynamics

Collaboration models between nurses and physicians are often ill‐defined and inconsistent, particularly regarding role delineation during ultrasound‐guided placement. The lack of clear, scenario‐specific guidelines (e.g., routine vs. complex cases) leads to inefficiency, delays, and frustration.

“The current collaboration is haphazard. Nurses typically handle routine placements independently without physician involvement. However, when encountering a difficult case and requesting physician assistance, they may be unwilling or unavailable due to other commitments. This creates bottlenecks and compromises timely care.” (N7)

Within the nursing team, the roles and responsibilities of nurses with different seniority levels and ultrasound certifications are not clearly defined. This results in underutilization of skilled nurses and prevents effective skill transfer to less experienced staff.

“Collaboration between nurses of different seniority levels lacks clarity and is often chaotic. Senior nurses who possess the necessary certification tend to perform the procedure, while junior nurses, who need the learning opportunity, are sidelined and unable to gain hands‐on experience. This hinders team development and skill dissemination.” (N8)

## 4. Discussion

This study identified multidimensional factors influencing the clinical adoption of POCUS through semistructured interviews with 12 healthcare providers in the NICU. Thematic analysis yielded six primary facilitators and six primary barriers. The six facilitators included adequate resource allocation and equipment availability; verifiable safety and clinical efficacy; intrinsic motivation and professional development needs; supportive and innovative work environment; strong management and leadership support; and clear pathways for process optimization. The six barriers were deficiencies in competency development systems; complexity of neonatal clinical presentations; lack of institutional policies and standardized procedures; sociocultural and cognitive resistance; perceived legal and ethical risks; and unclear team collaboration mechanisms. Strengthening existing facilitators and addressing objective barriers can help promote the application of POCUS in gastric tube placement in preterm infants.

### 4.1. Enhancing Equipment Usability and Increasing Awareness of POCUS’ Utility Among Relevant Personnel are Fundamental Prerequisites for Advancing Technology Implementation

Many participants highlighted factors such as device accessibility, portability, ability to produce clear images, and high compatibility with pediatric patients as facilitators for POCUS adoption. This finding aligned with the results of Luciani’s study [15]. Davis’s Technology Acceptance Model [16] has highlighted the crucial role of perceived ease of use. It is a key factor in healthcare professionals’ acceptance of a new technology for clinical application [17]. The number of POCUS devices, the frequency of updates and replacements, image clarity, and ease of use all influence healthcare professionals’ willingness to use the technology in clinical practice. Therefore, department leaders should seek to secure internal funding to provide a solid resource foundation for the implementation of this technology. Additionally, some nurses emphasized that perceived safety and clinical efficacy of POCUS were major drivers for implementing the technology. However, some nurses noted skepticism toward the technology among certain physicians and family members, which, to some extent, hindered its widespread implementation. The usefulness of perception technology is also a key factor in ensuring the successful implementation of new technologies in clinical practice [18]. Thought alignment refers to the consistency in understanding among implementers regarding the significance and value of new technology implementation, which is the first step in the action generation mechanism of new technology adoption [19]. Therefore, nursing managers should first reach consensus with department heads and actively organize educational lectures within the department on the application of ultrasound for the safety of gastric tube placement in preterm infants to enhance healthcare professionals’ understanding. Additionally, frontline nurses and doctors should strengthen education for parents to help them understand the safety and effectiveness of the technology and obtain their support and recognition.

### 4.2. Stimulating Self‐Driven Innovation Capabilities, Fostering a Conducive Innovation Environment, and Strengthening Leadership Support are Vital Forces for the Application of Innovative Technologies

Interview findings revealed that some nurses possess strong self‐motivation to implement new technologies, while others indicated that a positive learning atmosphere among colleagues and strong support from leadership serve as driving forces for their continuous learning. This aligned with the research findings of van der Weide et al. [20]. Innovative self‐efficacy, organizational innovation climate, and organizational support are protective factors for nurses’ innovative behavior [21–23]. Nurses with high self‐innovation efficacy can overcome challenges in applying technology. A high level of innovation climate and strong leadership support provide psychological safety for nurses’ innovative behaviors. Therefore, managers should cultivate and maintain nurses’ enthusiasm for work by providing a supportive work environment and recognizing and rewarding innovative achievements. Goals should be set based on individuals’ different goal‐oriented types to promote nurses’ commitment to their work and drive the application of innovative technologies in clinical practice.

### 4.3. Optimizing the Training System Will Help Promote the Further Application of POCUS

In this study, several participants noted that the training content related to using POCUS to confirm the position of gastric tubes was insufficiently comprehensive and lacked specificity. Additionally, issues such as high training costs affecting participation rates were identified. This aligned with the findings of Zeleke et al. [24], which identified the lack of appropriate training and professional knowledge among nurses as the primary obstacle to implementing nursing processes. The reason lies in the fact that critical care ultrasound has traditionally been applied primarily in adult ICUs and targeted at physicians. Only in recent years has it gradually been attempted for use in the neonatal population [25], with main practice areas involving neonatal lungs, necrotizing enterocolitis (NEC), and neurological disorders [26–28]. However, research on its application in gastric tube placement for preterm infants remains scarce. Furthermore, critical care ultrasound training programs lack specialty‐specific segmentation, and neonatal ultrasound training content is considerably limited. Additionally, as clinical ultrasound procedures remain predominantly physician‐led, training materials lack guidance tailored for nurses. In the clinical practice of applying POCUS for gastric tube placement in preterm infants, unique physiological characteristics (such as esophageal stenosis, extremely low birth weight, and poor thermoregulation), along with potential complications like hepatosplenomegaly, gastrointestinal distension, and fasting conditions, increase the challenge of implementation [25]. However, this critical training content closely related to preterm infant practice remains to be developed. Therefore, it is recommended that nursing managers take the lead in collaborating with multidisciplinary experts from departments such as intensive care medicine and ultrasound to develop a comprehensive, clinical practice issues‐based training program for nursing practices based on ultrasound confirmation of gastric tube placement in preterm infants, thereby enhancing nurses’ ability to master and apply this technique more comprehensively in clinical settings. Additionally, tiered management has been recognized as a key strategy for improving the overall quality of the nursing workforce. Optimizing the tiered training process for nurses and ensuring that training content aligns with job requirements can significantly improve training efficiency and nurses’ core competencies [29]. The training program should include assessment tools to evaluate nurses’ competency levels and divide learning modes based on tier and competency level to enhance training effectiveness. Regarding training costs, nursing leaders should strive to coordinate and communicate with hospital nursing management departments or their own departments to secure funding support, thereby reducing training costs for frontline nurses and increasing participation rates.

### 4.4. Strengthening Ethical Risk Management and Legal Compliance Is Essential for the Sustainable Promotion of POCUS

Participants in this study consistently expressed profound concerns about potential legal and ethical risks. A deeper analysis reveals three interconnected dimensions: First, there exists a standardization dilemma in the certification of operational qualifications [30]. This reveals an ambiguous gray area between “training completion” and “formal operational authorization.” The absence of an objective competency‐based certification system endorsed by institutions or professional associations leaves nurses’ operational qualifications lacking unified, legitimate grounds. In the event of adverse incidents, this ambiguity in qualifications exposes both the individual operator and their institution to significant legal risks. Second, the storage and management of ultrasound images raise substantial evidentiary and privacy concerns. On the one hand, the legal evidentiary status of POCUS images—as records of medical procedures and diagnostic processes—remains undefined. Arbitrary deletion could sever critical evidence chains in medical disputes. On the other hand, images containing pediatric patients’ anatomical structures constitute sensitive personal information. The absence of secure archiving systems exposes patients’ imaging privacy to leakage risks, directly violating the medical ethics’ confidentiality principle. Finally, ambiguous liability determination in medical disputes remains a core obstacle to implementation. When nurses independently perform and interpret POCUS, the boundaries between their personal responsibility, physicians’ supervisory duty, and hospital organizational accountability become blurred. Without clear protocols delineating individual execution responsibility, professional oversight responsibility, and institutional safeguarding responsibility, nurses may avoid performing procedures due to fear of disproportionate medical–legal liability. Therefore, it is recommended to establish a multidisciplinary working group comprising the Joint Medical Office, Critical Care Medicine Department, Ultrasound Department, and legal counsel. Its core mission is to develop a standardized training curriculum and objective competency assessment criteria for “the application of critical care ultrasound in gastric tube placement for preterm infants,” supplemented by expert consultation to ensure the standards’ efficacy. This system employs competency‐based certification to ensure that healthcare providers authorized for independent operation have not only completed theoretical training but also passed rigorous skill assessments through simulation and clinical practice. This provides objective, traceable evidence for qualification recognition. Second, medical institutions must establish compliant medical image management and privacy protection protocols, promptly integrating POCUS images into electronic health record systems for management. Establish clear policies specifying which key images with legal evidentiary value (e.g., “cloud sign” images confirming location) must be stored and archived, along with uniform retention periods. Technologically, ensure access controls and data encryption within the image management system to rigorously protect pediatric imaging privacy. This initiative not only strengthens the medical evidence chain but also provides critical support for subsequent quality reviews, teaching research, and addressing potential medical disputes. Furthermore, clearly define responsibility boundaries and liability exemptions within interdisciplinary collaboration. Specify the operational scopes for healthcare professionals at different certification levels (e.g., primary or advanced) to provide frontline practitioners with the necessary “psychological safety,” encouraging bold yet compliant practice within established protocols.

## 5. Strengths and Limitations

The strength of this study lies in the fact that it is one of the few studies that examines, from a qualitative perspective, healthcare professionals’ views on the barriers and facilitators associated with the use of ultrasound to determine the placement of gastric tubes in preterm infants. Additionally, we collected perspectives from three groups: frontline nurses, frontline physicians, and nursing leaders, in order to obtain a more comprehensive understanding.

There are still some limitations to this study. The findings of this study primarily stem from the experience of a single top‐tier tertiary hospital. While maximum variation sampling was employed to capture diversity within this internal perspective, caution is warranted when extrapolating results to healthcare institutions with differing resource levels, geographic locations, or cultural contexts. Future research should validate these findings in more diverse, multicenter settings. Furthermore, while this study has explored barriers and facilitators to POCUS implementation within the healthcare system from multiple internal healthcare provider perspectives, the role of parents of premature infants in the NICU holds significant importance in clinical practice. Future research could specifically examine the impact of family perspectives on POCUS adoption, which would provide valuable complementarity to the findings of this study.

## 6. Conclusion

This study explored the main factors hindering and promoting the implementation of the POCUS application for gastric tube position confirmation from the perspectives of frontline nurses, frontline doctors, and nursing leaders. Participants pointed out that there were issues with insufficient protection and support in areas such as training systems, institutional regulations, cognitive levels, and team collaboration. To break through the identified bottlenecks, specific strategies and policies must be formulated based on the core obstacles. Hospital and departmental leaders should prioritize securing internal funding for equipment acquisition and establish structured education programs to demonstrate the clinical utility and enhance acceptance of POCUS. In addition, it is imperative to develop a standardized, tiered, and competency‐based training curriculum. And institutional financial support must be secured to cover training costs and ensure broad participation. Furthermore, a multidisciplinary working group should be established to draft comprehensive institutional regulations to clearly define operational qualifications and delineate responsibility boundaries between nurses and physicians.

## Funding

This research was funded by a grant from the Nursing Research Project of Peking Union Medical College Hospital (XHHLKY202209).

## Conflicts of Interest

The authors declare no conflicts of interest.

## Data Availability

Research data are not shared.
